# The Hymen

**Published:** 1888-10

**Authors:** E. S. McKee

**Affiliations:** Cincinnati, Ohio


### THE HYMEN.

By E. S. McKEE, M. D., CINCINNATI, OHIO.

In a clinical lecture delivered in the Gynecological Clinic, Medical College of Ohio, Cincinnati, the doctor said:

*Gentlemen*—It is my privilege to present to you to-day two cases which you will seldom see in this or any other clinic. You may be surprised when I tell you that in five years these two are the only cases of hymen which I have seen here. My experience in other gynecological clinics has not even been so fortunate. I well remember how Prof. Bandl, in Vienna, called a class of practitioners, and with great pride and satisfaction demonstrated a hymen in a young woman of 24 years. He dwelt upon it at great length, evidently believing that his listeners had seldom, if ever, seen such a membrane, and fearful lest they, possibly himself, would never again behold such a sight.

*Case 1.*—A young lady, aged 20, parents born in England, comes to us from Bellevue, in our neighboring State, Kentucky, where, it seems, women are not only fair but virtuous. She first came under my observation three years ago, February 26th, 1885. Her condition then was one of interest. She stated that her menses had been absent for four months; she was sick at the stomach of mornings, and she presented a bloated appearance, especially in the lower abdominal region, which gave rise to great suspicion of pregnancy. Indeed, such was the diagnosis of many of the students present, from inspection alone. She said that the menses had appeared at the age of thirteen, recurring regularly every four weeks, and they continued for one week. She complained of pain in both ovarian regions, dysuria and dyspeptic symptoms. Her mother was with her, an examination was asked and readily granted, which was an argument against pregnancy.

The abdomen was examined externally. No enlargement of the uterus could be made out, but there was a great deposit of fat within the abdominal walls. Preparatory to a digital examination, the inspection of the external genitals was made, as is proper in each new case, and a normally shaped hymen, semi-lunaris, was found just as you see here to-day. The hymen is one in which sexual intercourse could not be accomplished without rupture. Pregnancy was excluded and the diagnosis made, amenorrhœa from torpidity of the ovaries. She was put on iron, quinia and nux vomica, and in seven weeks her monthlies returned and have recurred with fair regularity ever since. She occasionally comes to the clinic for relief from dyspepsia or slight dysmenorrhœa. This hymen, which has thus been under observation in this clinic for three years, is the typical semi-lunar form.

*Case 2.*—A young lady, aged 19, of German parentage and a resident of this wicked city. She first came here ten days ago complaining of dysmenorrhœa. Her menses appeared at the age of fourteen and have been regular as to time, amount and duration. She seems to have a dysmenorrhœa which is obstructive in its nature. She has improved under bromide of potassium. She readily consented to an examination and inspection; discovered the hymen circularis which you see here. This is the next most frequent form of the hymen semi-lunaris, but this is hardly as fair a specimen as the other. This is also a hymen which would not permit of coition without rupture.

